# A comparative machine learning study of schizophrenia biomarkers derived from functional connectivity

**DOI:** 10.1038/s41598-024-84152-2

**Published:** 2025-01-22

**Authors:** Victoria Shevchenko, R. Austin Benn, Robert Scholz, Wei Wei, Carla Pallavicini, Ulysse Klatzmann, Francesco Alberti, Theodore D. Satterthwaite, Demian Wassermann, Pierre-Louis Bazin, Daniel S. Margulies

**Affiliations:** 1 Cognitive Neuroanatomy Lab, INCC UMR 8002, CNRS, Université Paris Cité, Paris, France; 2 Wellcome Centre for Integrative Neuroimaging, Nuffield Department of Clinical Neurosciences, FMRIB Centre, University of Oxford, Oxford, UK; 3 MIND Team, Inria Saclay, Université Paris-Saclay, Palaiseau, France; 4 Neurospin, CEA, Gif-Sur-Yvette, France; 5 Max Planck School of Cognition, Leipzig, Germany; 6 Wilhelm Wundt Institute for Psychology, Leipzig University, Leipzig, Germany; 7 National Scientific and Technical Research Council (CONICET), Buenos Aires, Argentina; 8 Department of Physics, Institute of Applied and Interdisciplinary Physics, University of Buenos Aires, Buenos Aires, Argentina; 9 University of Pennsylvania, Perelman School of Medicine, Philadelphia, PA USA; 10 Full Brain Picture Analytics, Leiden, The Netherlands

**Keywords:** Biomarkers, Machine learning, Schizophrenia

## Abstract

Functional connectivity holds promise as a biomarker of schizophrenia. Yet, the high dimensionality of predictive models trained on functional connectomes, combined with small sample sizes in clinical research, increases the risk of overfitting. Recently, low-dimensional representations of the connectome such as macroscale cortical gradients and gradient dispersion have been proposed, with studies noting consistent gradient and dispersion differences in psychiatric conditions. However, it is unknown which of these derived measures has the highest predictive capacity and how they compare to raw functional connectivity specifically in the case of schizophrenia. Our study evaluates which connectome features derived from resting state functional MRI — functional connectivity, gradients, or gradient dispersion — best identify schizophrenia. To this end, we leveraged data of 936 individuals from three large open-access datasets: COBRE, LA5c, and SRPBS-1600. We developed a pipeline which allows us to aggregate over a million different features and assess their predictive potential in a single, computationally efficient experiment. We selected top 1% of features with the largest permutation feature importance and trained 13 classifiers on them using 10-fold cross-validation. Our findings indicate that functional connectivity outperforms its low-dimensional derivatives such as cortical gradients and gradient dispersion in identifying schizophrenia (Mann–Whitney test conducted on test accuracy: connectivity vs. 1st gradient: U = 142, *p* < 0.003; connectivity vs. neighborhood dispersion: U = 141, *p* = 0.004). Additionally, we demonstrated that the edges which contribute the most to classification performance are the ones connecting primary sensory regions. Functional connectivity within the primary sensory regions showed the highest discrimination capabilities between subjects with schizophrenia and neurotypical controls. These findings along with the feature selection pipeline proposed here will facilitate future inquiries into the prediction of schizophrenia subtypes and transdiagnostic phenomena.

**Supplementary Information:**

The online version contains supplementary material available at 10.1038/s41598-024-84152-2.

## Introduction

Functional connectivity holds promise as a potential biomarker for schizophrenia^1–5^, as evidenced by a robust body of fMRI literature that highlights distinct functional profiles between people with schizophrenia and neurotypical individuals. Prior studies have reported lower connectivity across regions, reduced small-worldness of the resting state networks, and lower functional network segregation^5–7^. However, when the goal is to predict clinical status using the entire functional connectome as features for model training, the resulting model becomes high-dimensional and overly complex. This high dimensionality, coupled with small sample sizes in clinical research, increases the risk of overfitting^8–10^.

Recently, low-dimensional representations of the connectome such as macroscale cortical gradients^11,12^ and gradient dispersion^13,14^ have been proposed. The gradients are derived from functional connectivity matrices through dimensionality reduction algorithms such as principal component analysis (PCA) or diffusion map embedding. The aim of this computation is to maximize the cumulative amount of variance explained by the resulting components. The first component (also known as the *principal gradient*) explains the largest fraction of connectivity variance. It reflects the functional hierarchy of the cortex^11,12,15^, spanning from the primary sensory (*unimodal*) regions to higher-order (*transmodal*) regions. The principal gradient has been demonstrated to be consistent across individuals^16,17^.

Dong et al.^18^ revealed that the principal gradient is contracted in schizophrenia. That is, the primary sensory regions were reported to be closer to the higher-order regions in terms of their functional connectivity profile, as indicated by their principal gradient values. This finding indicates lower functional differentiation between uni- and transmodal regions. In other words, the primary sensory and higher-order processing regions are more functionally similar in subjects with schizophrenia compared to neurotypical individuals. In addition, Holmes et al.^19^ reported significant differences in the second gradient which spans the hierarchy of primary sensory areas. Based on the gradient framework, other neurodevelopmental, psychiatric and neurodegenerative disorders have been reported to manifest changes in the cortical functional hierarchy^14,16,20–28^.

As an extension of the gradient framework, gradient dispersion further quantifies the density of local connectivity between or within clusters of areas. This density characterizes the extent to which the areas are functionally segregated or integrated. Specifically, higher dispersion would indicate higher functional segregation within a cluster of regions, whereas lower dispersion would mean higher functional integration^13,27^. Thus, gradient dispersion characterizes functional differentiation across the cortical hierarchy: high dispersion of a cluster of regions points to their functional dissimilarity, placing them farther apart along the functional hierarchy of the cortex. Gradient dispersion is of interest to us since changes in the cortical hierarchy have been reported to be idiosyncratic to schizophrenia^1,18,29^. However, gradient dispersion has not yet been tested as a potential predictor of schizophrenia.

**Fig. 1:**
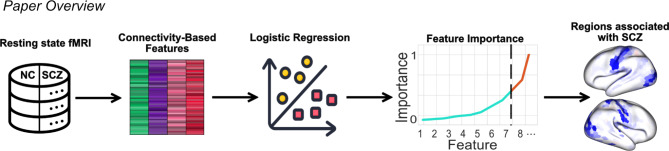
Overview of the methods and main outcome of the paper. Schematic images: Flaticon.com. NC: neurotypical controls, SCZ: individuals with schizophrenia.

Overall, there is a growing interest in the macroscale cortical hierarchy as a predictor of various phenotypes, be it age^13,30^, psychosis^18,19^, or task performance^31,32^. However, it is unknown if cortical gradients or gradient dispersion can discriminate between people with schizophrenia and neurotypical individuals, or if they outperform raw connectivity. Given this gap and the recent prominence of the gradient framework, we selected gradients and gradient dispersion as biomarker candidates for this study, in addition to raw functional connectivity. Here, we attempt to identify the features with the largest biomarker potential from a large set of features including the aforementioned measures. In addition, we explore the impact of the number of features on the choice of classifier. We also seek to address the question germane to neuroscience and computational psychiatry: when one has a limited number of subjects and a disproportionately rich set of independent variables, how does one justify the choice of features? To this end, we develop a pipeline based on permutation feature importance which allows us to assess the predictive power of all features at once in a computationally efficient manner (Fig. 1). Additionally, we elaborate on putative functional underpinnings of schizophrenia based on the features with the highest predictive potential.

## Methods

### Data and preprocessing

The present study’s sample was derived from three publicly available datasets: COBRE^33^, LA5c study from UCLA Consortium for Neuropsychiatric Phenomics^34^, and the SRPBS-1600 multidisorder MRI dataset^35^. All data were acquired in accordance with the Declaration of Helsinki. For each dataset, the acquisition was approved by the local institutional review board (IRB). Informed consent was obtained from all subjects and/or their legal guardian(s) to the collection of imaging and behavioral data. The links to the datasets are provided in the data availability statement below.

The initial cohort for our investigation consisted of 996 individuals; subsequently, 60 subjects were excluded from analysis due to substantial motion artifacts (mean framewise displacement (FD) > 0.5 mm). The analyzed sample comprised 248 subjects with schizophrenia (SCZ) and 688 neurotypical controls (NC). We conducted a Chi-squared test for sex and Mann–Whitney U tests for age and mean FD. Some datasets were significantly different in terms of sex, age and mean FD distributions (see Table 1). We address this limitation by including these variables as covariates in all analyses. The scanning parameters of each dataset are reported in Supplementary Table 1.

**Table 1:** Demographic statistics of the sample.

Site	Group	N	Mean age (SD)	*U*_*AGE*_	N female (N male)	*χ*^2^_SEX_	Mean FD (SD) (mm)	*U*_*FD*_
COBRE	NC	81	37.9 (11.9)	2598	23 (58)	1.87	0.25 (0.1)	**1860**
	SCZ	59	36.4 (13.2)		10 (49)		0.29 (0.1)	
LA5c	NC	102	30.6 (8.3)	**1456**	49 (53)	**7.62**	0.21 (0.07)	**1232**
	SCZ	45	36.3 (8.9)		10 (35)		0.21 (0.09)	
KTT*	NC	75	28.9 (9)	**879**	27 (48)	0.39	0.1 (0.04)	**1260**
	SCZ	46	37.6 (9.7)		20 (26)		0.13 (0.05)	
KUT*	NC	159	36.5 (13.6)	**2497**	66 (93)	1.51	0.15 (0.07)	3112
	SCZ	43	42.1 (10.4)		23 (20)		0.15 (0.07)	
SWA*	NC	101	28.4 (7.9)	**205**	15 (86)	0.11	0.15 (0.07)	**472**
	SCZ	19	42.9 (8.4)		4 (15)		0.22 (0.09)	
UTO*	NC	170	35.6 (17.5)	3194	92 (78)	**4.34**	0.12 (0.07)	**2810**
	SCZ	36	31.4 (10.3)		12 (24)		0.13 (0.06)	
All data	NC	688	33.5 (13.2)	**65103**	272 (416)	**4.34**	0.15 (0.08)	**62110**
	SCZ	248	37.4 (11.1)		79 (169)		0.19 (0.1)	

Significant comparisons are in bold and underlined. *These datasets are part of a larger dataset, SPRBS-1600. FD: framewise displacement.

Preprocessing of MRI data was done using fMRIPrep 20.2.1^36^ which is based on Nipype 1.5.1^37^ (Supplementary Methods 1). The preprocessed BOLD time series were parcellated with the Schaefer parcellation (1000 parcels, 7 Yeo networks)^38^. Then, we computed a connectivity matrix (Pearson correlation) for each subject.

### Macroscale cortical gradients

**Fig. 2:**
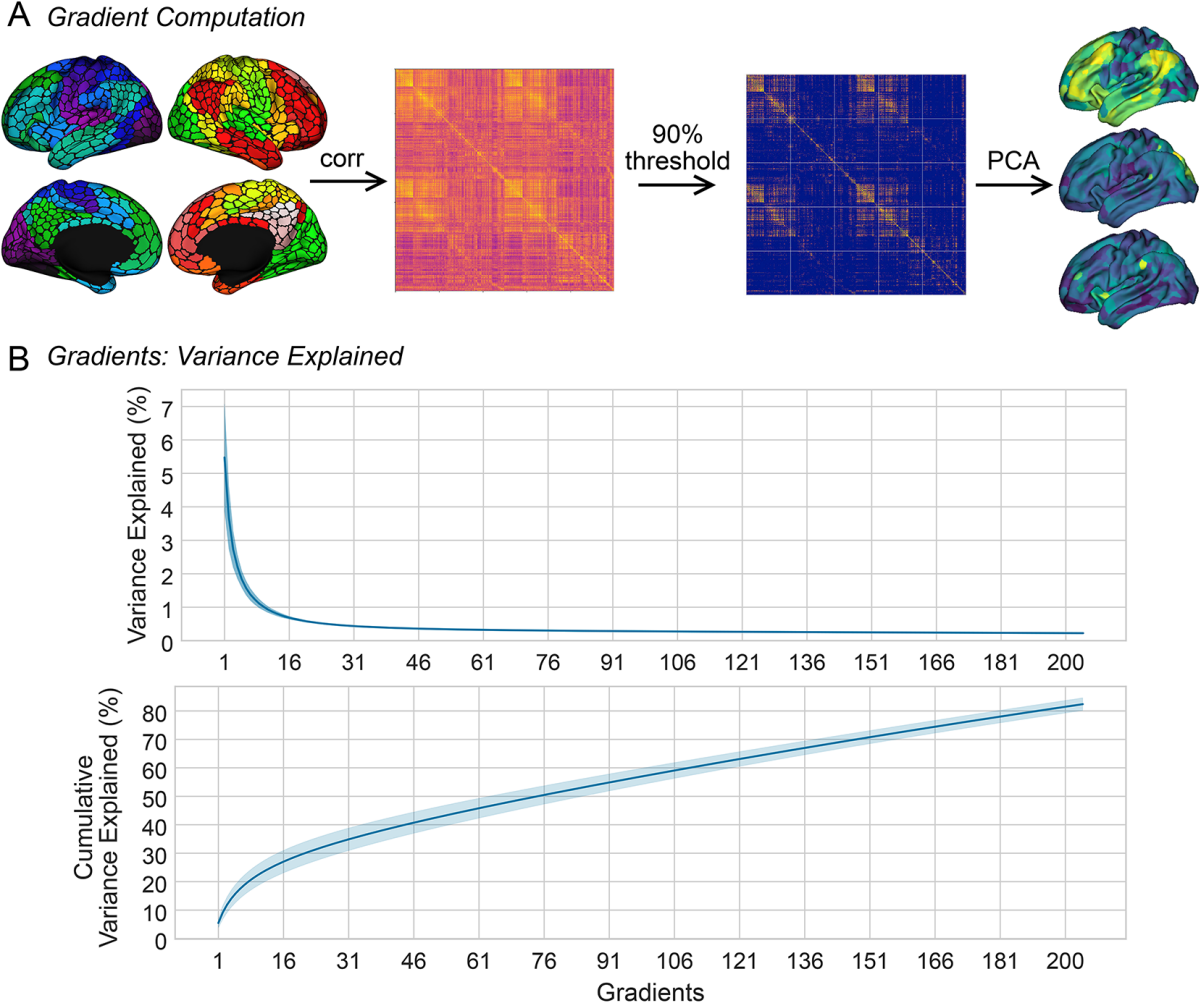
(**A**) Parcel-wise time series (Schaefer atlas, 1000 parcels, 7 Yeo networks^38^) of each subject were correlated to produce a 1000 × 1000 connectivity matrix. Principal component analysis (PCA) was applied to the thresholded matrix to extract 200 gradients. (**B**) Variance explained by 200 gradients, mean across subjects ± 1 s.d.

For each subject, we computed cortical gradients by applying PCA to the Fisher z-transformed and thresholded connectivity matrix (Fig. 2A). Hong et al.^16^ showed that PCA, when applied to thresholded connectivity matrices, yields more reliable gradients compared to the other dimensionality reduction techniques frequently featured in the gradient literature^12,39,40^. We thresholded the connectivity matrices by discarding 90% of the lowest correlation values including negative values. We used Procrustes alignment to align the gradients of all subjects^41^. To avoid introducing dataset-specific bias to the alignment, we used the gradients computed from the group connectivity matrix of the Human Connectome Project (HCP)^42^ as reference gradients. Figure 2B displays mean variance explained across all subjects for 200 gradients. On average, the principal gradient accounted for ~ 6% of variance of thresholded connectivity matrices. Collectively, 200 gradients accounted for ~ 80% of variance. Given that 1000 gradients cumulatively explained all variance (Supplementary Fig. 1), we deemed 200 gradients computationally optimal as 20% of gradient values in this case account for 80% of variance.

### Centroid (gradient) dispersion

Gradient dispersion was first introduced by Bethlehem et al.^13^ and has since been employed in other studies^14,19,43^. Prior investigations, akin to the present study, used gradient dispersion to operationalize functional modularity—i.e., functional (dis)similarity—across the cortex. However, these methods necessitated the identification of centroids of networks for which dispersion was computed relative to the regions encompassed within the network^13^. Thus, dispersion quantifies functional modularity within a network or between networks.

Following this procedure, we computed within- and between-network dispersion for seven Yeo networks^65^ in the 3-dimensional gradient space constituted by the first three principal components of functional connectivity. Within-network dispersion was quantified as the sum of squares of Euclidean distances from the centroid of the network to the rest of its regions. For the gradient values belonging to a given network, the centroid was defined as three median values of the first three cortical gradients, as in Bethlehem et al.^13^; the position of the centroid in the 3D latent gradient space is defined by these three values. Thus, given a network *k* consisting of *P* parcels with each parcel *p* having gradient values *g*, within-network centroid dispersion is computed as follows:

1 $${\text{Within - network}}\;{\text{CD}}_{{\text{k}}} = \mathop \sum \limits_{{{\text{p}} = 1}}^{{\text{P}}} \left\| {{\text{g}}_{{{\text{p}},{\text{k}}}} - {\text{median}}\left( {{\text{G}}_{{\text{k}}} } \right)} \right\|^{2}$$

where *G*_*k*_ is a matrix of all gradient values of all parcels in network *k*, ${\text{g}}_{\text{p},\text{k}}\in {\mathbb{R}}^{3}$.

Between-network dispersion was defined as the Euclidean distance between the network centroids as follows:

2 $${\text{Between - network}}\;{\text{CD}}_{{{\text{kl}}}} = \left\| {{\text{median}}\left( {{\text{G}}_{{\text{k}}} } \right) - {\text{median}}\left( {{\text{G}}_{{\text{l}}} } \right)} \right\|$$

where *G*_*k*_ and *G*_*l*_ are matrices containing all gradient values of all parcels in networks *k* and *l*.

**Fig. 3:**
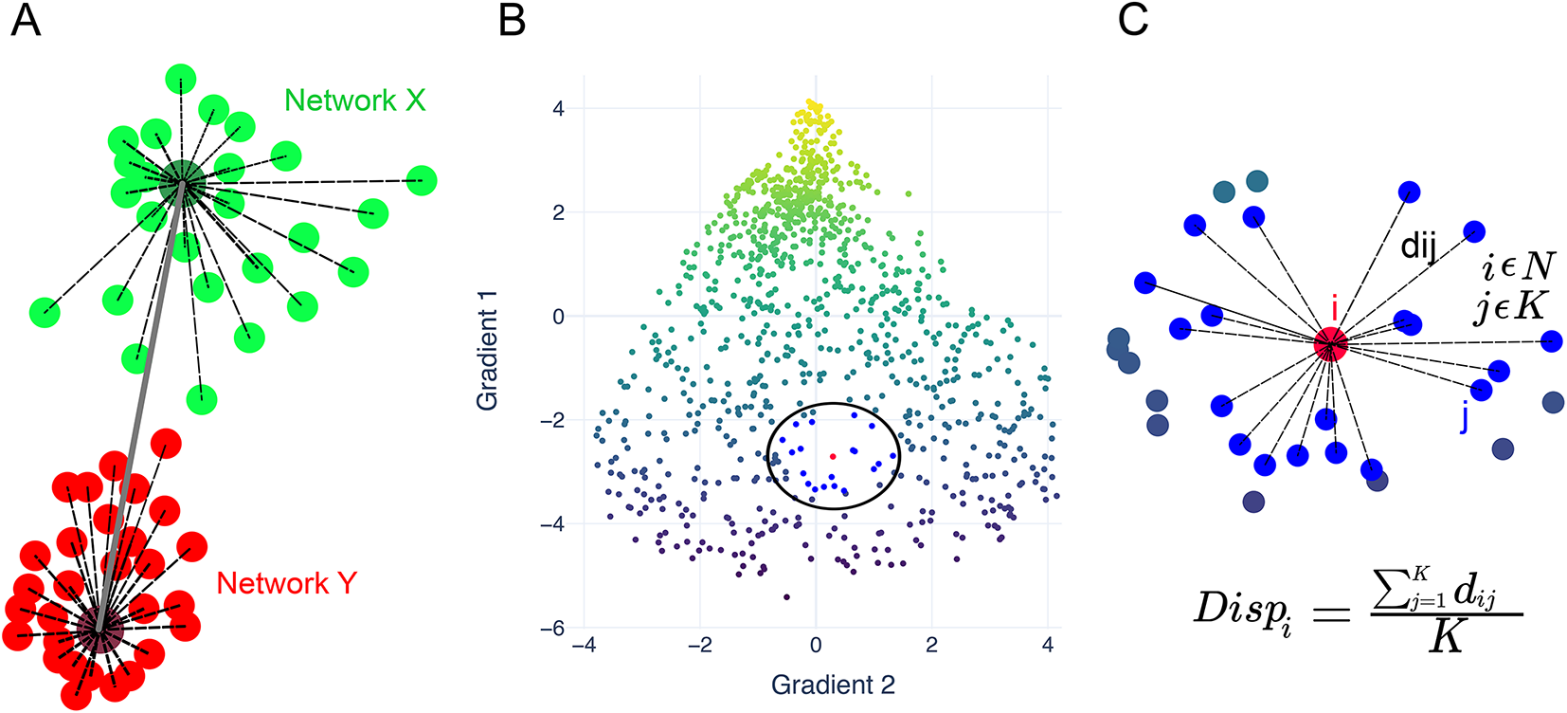
Illustration of the methods used to compute gradient dispersion. (**A**) Centroid dispersion. The sum of squares of distances between the centroid of a network and its regions (black dashed lines) quantifies within-network dispersion. The distance between the centroids of networks quantifies between-network dispersion. (**B**) Neighborhood dispersion. In a multidimensional gradient embedding, for a given region (red) K nearest neighbors are identified (blue). These regions are shown within the black circle. (**C**) Neighborhood dispersion of a given region *i* is the mean distance between said region and its K closest neighbors. The same operation is done for every region (N regions = 1000).

As a result of these computations, centroid dispersion amounted to 28 values per subject: 7 values for within-network and 21 values for between-network dispersion. Centroid dispersion is schematically illustrated in Fig. 3A.

### Neighborhood (gradient) dispersion

Centroid dispersion can vary depending on how the networks are delineated (e.g., if a different network parcellation is used). Hence, we computed neighborhood dispersion with the aim to circumvent this potential confounding factor. Specifically, we calculated dispersion for individual regions which enabled us to maintain the spatial resolution congruent with the gradients (1000 regions per measure).

To compute neighborhood dispersion for every region, we identified K closest neighboring regions (Fig. 3B,C) via the K-Nearest Neighbors (KNN) algorithm. Then, we computed the mean Euclidean distance between the focal region and its designated neighboring regions in the gradient space. The resulting value quantified dispersion for the focal region. Neighborhood dispersion was computed for combinations of gradients spanning from 1 to 200.

Unlike previous investigations where the primary source of variability in dispersion originated from network delineation, our study has its own unique challenge in determining the number of the nearest neighbors (K). To address this issue, we included all dispersion values computed based on a range of nearest neighbors from 10 to 170 with a step size of 40. We chose this step size to reduce the computational load of this operation, i.e., we did not perform any optimization for K. Mathematically, neighborhood dispersion can be formulated as follows:

3 $$ND_{f} \left( K \right) = \frac{1}{K}\mathop \sum \limits_{j = 1}^{K} \left\| {x_{{\text{f}}} - x_{j} } \right\|$$

where $K\in [10, 50, 110, 140, 170]$ (number of neighbors), ${x}_{f},{x}_{j}\in {\mathbb{R}}^{d}$ with ${x}_{f}$ being the gradient values of the focal region and ${x}_{j}$ being the gradient values of its *j*^*th*^ neighbor in a *d*-dimensional gradient space ($d\in \left[1:200\right]$).

Basing the calculation of dispersion on differing sets of gradients (each including up to 200 gradients), we derived 1000 × 200 × 5 = 1,000,000 neighborhood dispersion values, which we then used as input to our analytic workflow (along with flattened connectivity matrices and gradients).

In summary, *centroid dispersion* is computed based on the Yeo networks and the first three gradients as in Bethlehem et al.^13^ (Eqs. 1, 2). Conversely, *neighborhood dispersion* is computed using a range of the nearest neighbors and combinations of gradients from 1 to 200 (Eq. 3).

### Analytic workflow

**Fig. 4:**
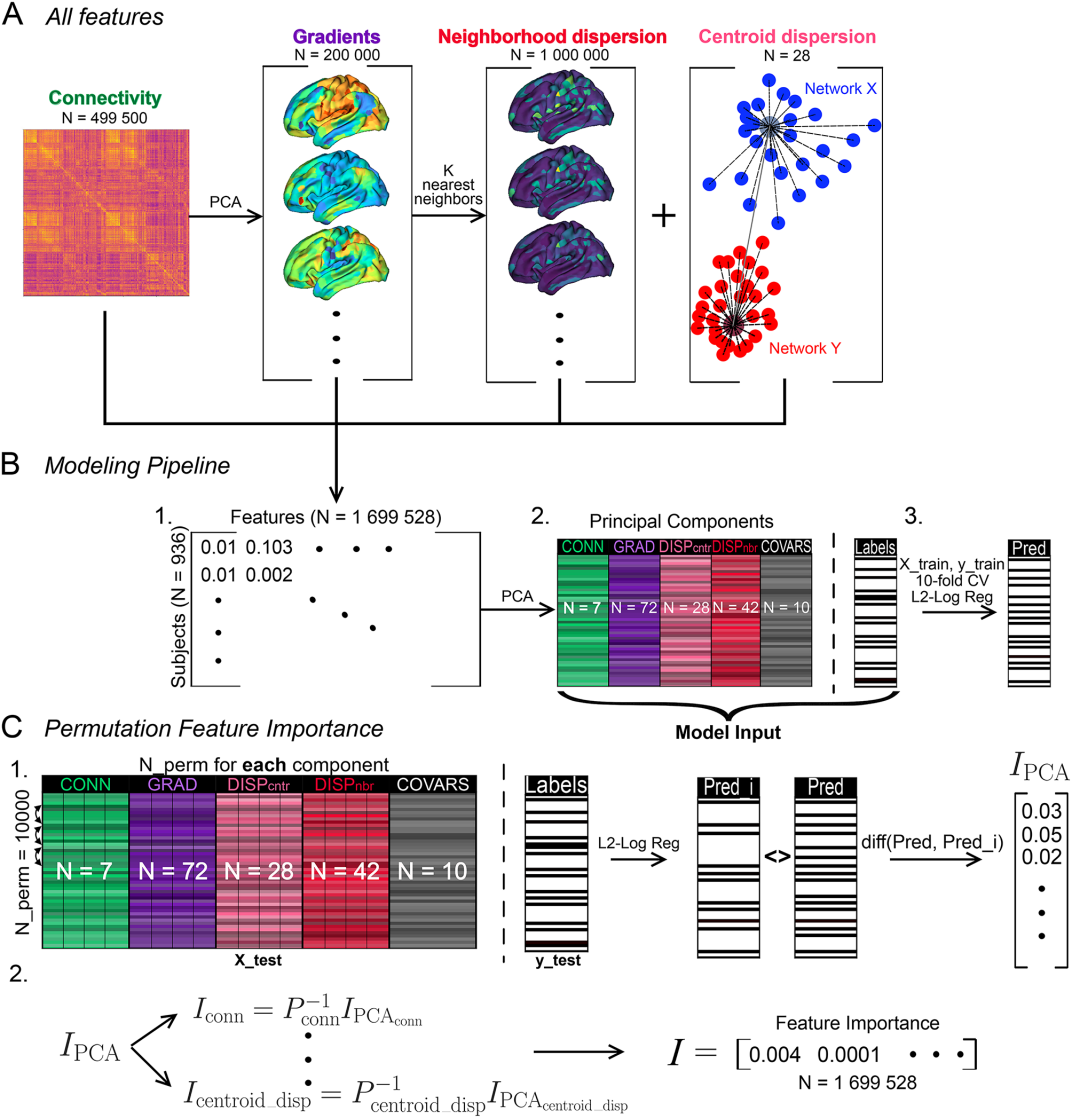
(**A**) The types of predictors tested in this work (left to right): connectivity matrices (vectorized), macroscale cortical gradients, neighborhood, and centroid dispersion. (**B**) All four types of features were concatenated together (2) and decomposed using group PCA (2) (each feature group is decomposed separately). The resulting dataset, along with covariates, was divided into the train and holdout set; 10-fold cross-validation (CV) was used to assess the performance of L2-regularized logistic regression on the PCA dataset (3). (**C**) Permutation component importance was computed for each component using the holdout set (1). For each feature type, component importance was inverse transformed to obtain feature importance (2). COVARS: covariates (age, sex, framewise displacement, site), CONN: connectivity, CV: cross-validation, DISPcntr: centroid dispersion, DISPnbr: neighborhood dispersion, GRAD: cortical gradients, I_PCA_: component permutation importance, L2-Log Reg: L2-regularized logistic regression, PCA: principal component analysis.

The objective of our workflow was to identify the features with the largest predictive capacity from an extensive array of connectivity-based features. Our dataset included vectorized connectivity matrices (N = 499,500), 200 gradients (N = 200,000; 1000 values per gradient; 200 values per region), neighborhood dispersion (N = 1,000,000; 1000 values per region) and centroid dispersion (N = 28) (Fig. 4A). Since the number of candidate features is too large for every feature combination to be tested separately (for a fixed N_features_ = 1000: ${N}_{combs}= \frac{1699528!}{1000!\left(1699528-1000\right)!} \approx 3.95 * {10}^{3010}$), we designed a custom feature selection pipeline (Fig. 4). For each participant, we combined all features into one flat vector of size [1,699,528]. Then, we concatenated the vectors for all 936 participants, resulting in a matrix of size [936 × 1,699,528] (Fig. 4B, 1). Next, we applied PCA to each type of features separately and retained 20% of variance for each type (effectively compressing the feature dimension), except for the centroid dispersion for which all variance was included (Fig. 4B, 2). The aim of the decomposition was i. to retain the same amount of variance for all feature types, ii. to ensure that for each feature type more than one component is extracted when applying PCA, and iii. to alleviate the imbalance of the number of features available for each feature type (feature type ratio in the full feature matrix: 50:20:100:0.0028 and in the PCA feature matrix: 7 : 72 : 42 : 28). Thus, we retained 149 components in total (N_conn_ = 7, N_grad_ = 72, N_centroid_disp_ = 28, N_cortex_disp_ = 42; Fig. 4B, 2). The decomposed dataset was divided into the train (75% of participants) and holdout (25%) sets, with the ratio of N_SCZ_/N_NC_ = 0.35 in both.

The purpose of the following steps was to quantify the importance of each component for classification performance. We fitted an L2-regularized logistic regression on the train set (Fig. 4B, 3). Logistic regression was determined as the best model to compute component importance since the resulting coefficients are interpretable and the logic behind their computation is well understood. We used *permutation feature importance* (Fig. 4C, 1) as the measure of the contribution of each component and feature to classification performance. Initially conceived for random forests^44,45^, it allows us to estimate the importance of the features in a classifier-agnostic way using the holdout set. First, the classifier is trained on the train set and the baseline accuracy on the holdout set is obtained. Second, each feature of the holdout set is randomly shuffled N_perm_ times and at each shuffle the permutation accuracy is computed. Permutation feature importance is the difference between the baseline accuracy and the permutation accuracy at each permutation. This feature importance metric can be estimated for any classifier; it quantifies the extent to which classification performance deteriorates or improves as every feature is shuffled in the holdout set. The feature with the largest permutation importance contributes the most to classification performance (Fig. 4C, 1). We computed mean permutation importance for each component (N_perm_ = 10,000) and inverse transformed it based on the components’ projection matrices to obtain feature importance in the feature space (Fig. 4C, 2). Permutation feature importance enabled the selection of features for further assessment of their utility for classification.

### Classifier analysis

Finally, we assessed the predictive capacity of each feature type in a classifier-agnostic manner to prevent the results from being driven by the choice of a specific classifier. To this end, we selected the top 1% of features with the largest permutation feature importance and we trained and tested 13 distinct classifiers on them:

- Logistic regression (L2-regularized, LR).
- K-Neighbors Classifier (KN).
- Naïve Bayes (NB).
- Decision Tree Classifier (DT).
- Support Vector Machine (SVM).
- Ridge Classifier (Ridge).
- Random Forest Classifier (RF).
- Ada Boost Classifier (AB).
- Gradient Boosting Classifier (GB).
- Light Gradient Boosting Machine (LGB).
- Linear Discriminant Analysis (LDA).
- Extra Trees Classifier (ET).
- Quadratic Discriminant Analysis (QDA).

To verify that the difference in classification performance between feature types persists regardless of the number of features, we repeated this analysis for a range of 100 to 10,000 features with the largest permutation feature importance from each type.

For each feature count, we identified the best classifier based on its mean cross-validation (CV) accuracy across 10 folds. Next, we tested the best classifier on the holdout set. All data transformations were done within Scikit-Learn pipelines, i.e., they were performed separately on the test and holdout sets. The multi-classifier analysis was done using Pycaret (https://github.com/pycaret/pycaret). Age, sex, site and framewise displacement (FD) were always included as covariates.

For classifier performance, we report both accuracy and the F1-score. The F1-score, calculated using the Scikit-Learn package in Python, represents the harmonic mean of precision and recall. This metric is particularly useful in cases where class distribution is imbalanced, as it accounts for both false positives and false negatives. We report accuracy and F1-score for cross-validation (CV) folds and the held out sets. In CV, each fold consists of a training and validation set. Thus, CV metrics were computed on the validation set of each fold. For instance, mean CV accuracy is the mean across accuracies computed on validation sets of 10 folds. Regarding test performance, whenever we refer to test F1-score or accuracy, we refer to the performance on the subjects that the model has not seen during training, i.e., these subjects were not present in any of the 10 folds the model was trained and validated on.

The CV and test performance across all classifiers and feature subsets was compared to two baselines, namely:

1. Test performance of the logistic regression trained on the PCA dataset (all 149 components) on the hold-out set.
2. The dummy classifier which randomly picks the class for each sample. It is frequently used as a baseline in machine learning research^46,47^. Note that in this study the accuracy of the dummy classifier consistently remained at 73.7%. In contrast, the F1 score for this classifier is always 0, meaning that it does not differentiate between the two classes. These values constitute our chance reference. We also conducted the multi-classifier analysis for the principal components of the feature types (for the full ranking of the models trained on the components of features see Supplementary Table 2). Logistic regression was ranked 2^nd^ for accuracy and 1^st^ for F1-score.

### Localization of the best features in the brain: weighted degree centrality

The analyses described above allow us to identify the features exerting the largest influence on model predictions. However, it is also necessary to establish their links to specific brain areas. To this end, we conducted an additional exploratory analysis. Since raw connectivity was identified as the best feature type, this analysis was tailored for connectivity edges.

For three feature subsets including 500, 1000 and 5000 connectivity edges with the largest permutation feature importance, we computed weighted degree centrality (WDC). For each subset, the selected edges were transformed back to a 1000 × 1000 connectivity matrix and for each region the sum of edges was computed, each edge weighted by its correlation value. The computation of WDC can be mathematically formulated in what follows. Given a connectivity matrix *C*_*subj*_ of a specific subject, let *E*_*selected*_ be a set of indices of the edges with the highest permutation feature importance. In *C*_*subj*_, we mask the edges whose indices are not in *E*_*selected*_:

4 $$C_{subj, selected} = M \odot C_{subj} \; where \; M = \left\{ {\begin{array}{*{20}l} {1,} \quad {if \;\left( {i,j} \right) \in E_{selected} \; or \; \left( {j,i} \right) \in E_{selected} } \hfill \\ {0,} \quad {otherwise} \hfill \\ \end{array} } \right.$$

Next, for neurotypical and individuals with schizophrenia, we compute mean WDC for every region using the unmasked correlation values:

5 $$WDC_{group} \left( {C_{subj, selected} } \right) = \frac{1}{S}\mathop \sum \limits_{subj = 1}^{S} \mathop \sum \limits_{j = 1}^{1000} c_{i,j} ; \quad c_{i,j} \in C_{subj, selected}$$

where *S* is the number of subjects in the group. Of note, those edges which were not selected based on their feature importance ($\left(h,m\right) \notin {E}_{selected}$) were set to 0 in *C*_*subj,selected*_ as per Eq. 4. In summary, for each region, we sought to quantify its connectivity strength to the other regions given the selected edges.

## Results

### Permutation feature importance and classification performance

**Fig. 5:**
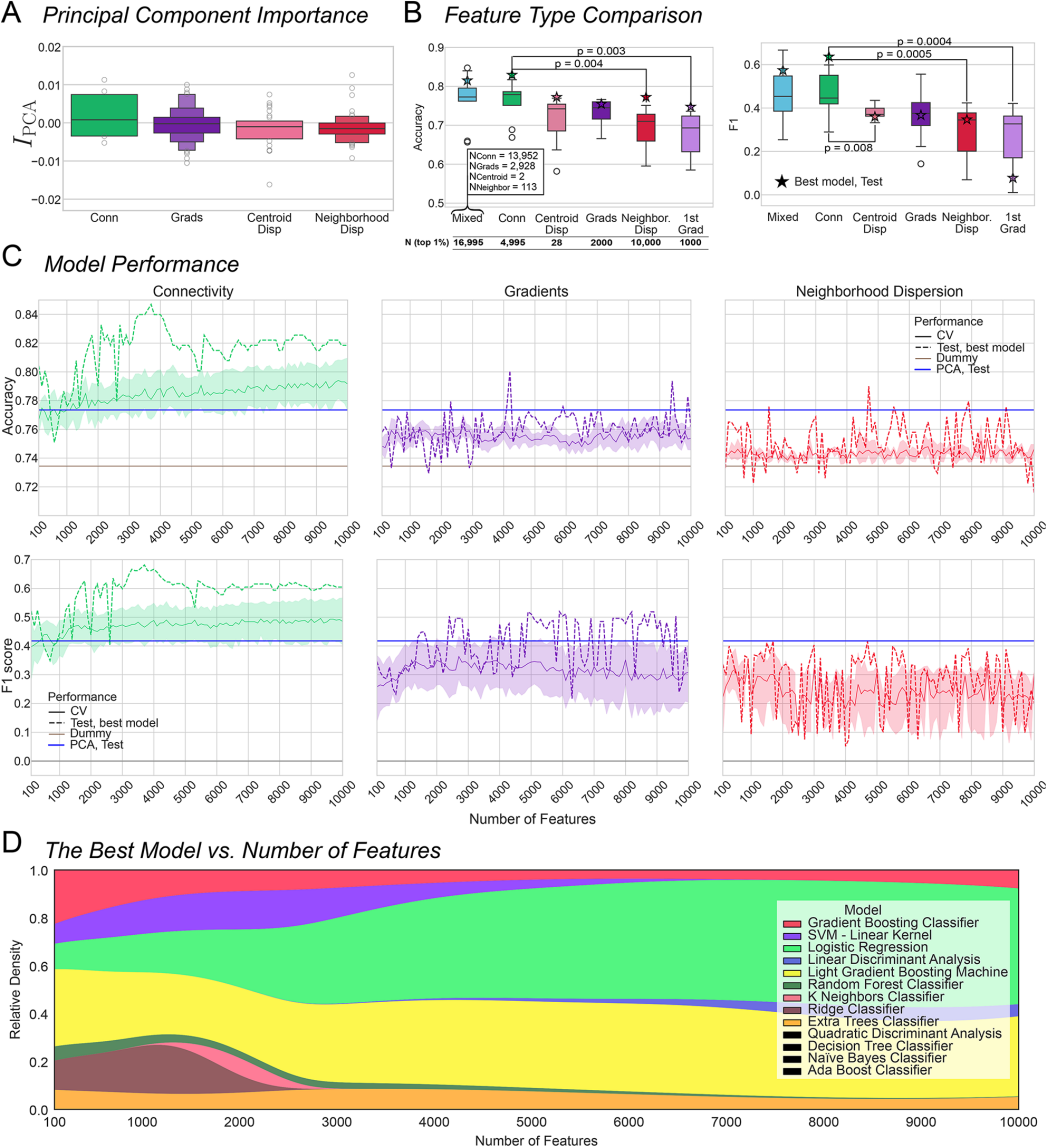
(**A**) Permutation importance across feature types. (**B**) Accuracy and F1 score across 13 classifiers (mean cross CV folds; dummy classifier was excluded) fit on top 1% best features from each feature type, the principal gradient, the 28 values of centroid dispersion and the top 1% best features from the whole feature set (mixed: the inset shows the number of features from each feature type that were included in the top 1%). *P*-values indicate significant difference as per Mann–Whitney U test, *α* ≤ 0.01 (connectivity vs. all: Bonferroni-corrected). The stars denote the performance of the best classifier as identified based on the mean accuracy across 10 CV folds. (**C**) Mean ± s.e.m. CV and test performance across classifiers for N features 100–10,000 for connectivity (left), gradients (middle), and neighborhood dispersion (right). Horizontal lines represent test performance of the logistic regression on all principal components (blue), and the performance of the dummy classifier (brown). The shading indicates s.e.m. (**D**) Relative density of fits where the corresponding classifier was identified as best. Larger area indicates that the corresponding classifier had the highest CV accuracy more often. The legend features all classifiers that were tested in this study; the classifiers in black were never identified as the best. CV: cross-validation; PCA: principal component analysis; SVM: support vector machine.

Upon visual inspection, we observed that the principal components of functional connectivity had the largest permutation feature importance, followed by gradients, centroid dispersion, and neighborhood dispersion (Fig. 5A). We sought to verify that permutation feature importance indeed reflects an advantage in classification performance regardless of classifier. To this end, we selected the top 1% of features with the largest permutation feature importance for each type and trained and tested 13 classifiers on them (Fig. 5). Connectivity outperformed the other feature types. This conclusion was supported by the Mann–Whitney U test (Fig. 5B, Table 2) for both accuracy and F1-score. To limit the number of comparisons, we only performed tests for connectivity vs. all other types which amounted to 5 tests for accuracy and F1-score. The results of all comparisons are displayed in Table 2.

**Table 2:** Results of Mann–Whitney U test.

Top 1%	Gradient 1	Neighborhood dispersion	Centroid dispersion	All gradients	All features	Metric
Connectivity	U = 142, *p* = 0.003*	U = 141, *p* = 0.004*	U = 128, *p* = 0.027	U = 127, *p* = 0.03	U = 83, *p* = 0.95	Accuracy
	U = 154, *p* = 0.0004*	U = 153, *p* = 0.0004*	U = 137, *p* = 0.008*	U = 126, *p* = 0.04	U = 85, *p* = 1.0	F1-score

We compared the performance of the top 1% of the most important features from connectivity to all the other types. Significant results are marked with an asterisk. The results were corrected for multiple comparisons (Bonferroni: α = 0.01). These results are also represented graphically in a boxplot in Fig. 5B.

Multi-classifier analyses further emphasized the superior predictive capacity of functional connectivity compared to the other feature types. Here we report CV and test accuracy and F1-score across all classifiers and for the best classifier (Fig. 5C). Connectivity edges with the largest permutation feature importance consistently outperformed logistic regression fitted on all feature components (Supplementary Table 2). The other feature types performed substantially worse.

In addition, we examined the impact of the number of best features on the choice of the best classifier (fits on all feature types are considered). Figure 5D illustrates the evolution of the best classifier with the increasing number of features as the change in relative density of instances where the classifiers were identified as best. Three main patterns can be noted. Firstly, several classifiers clearly performed better with N_features < 3000: GB, SVM, RF, KN and Ridge. Secondly, ET and LGB had a relatively stable winning rate across all feature subsets. Thirdly, LR and LDA had an increased performance when N_features > 3000. However, LDA rarely outperformed the other classifiers, whereas LR and LGB often emerged as optimal.

### The features with the largest permutation feature importance

**Fig. 6:**
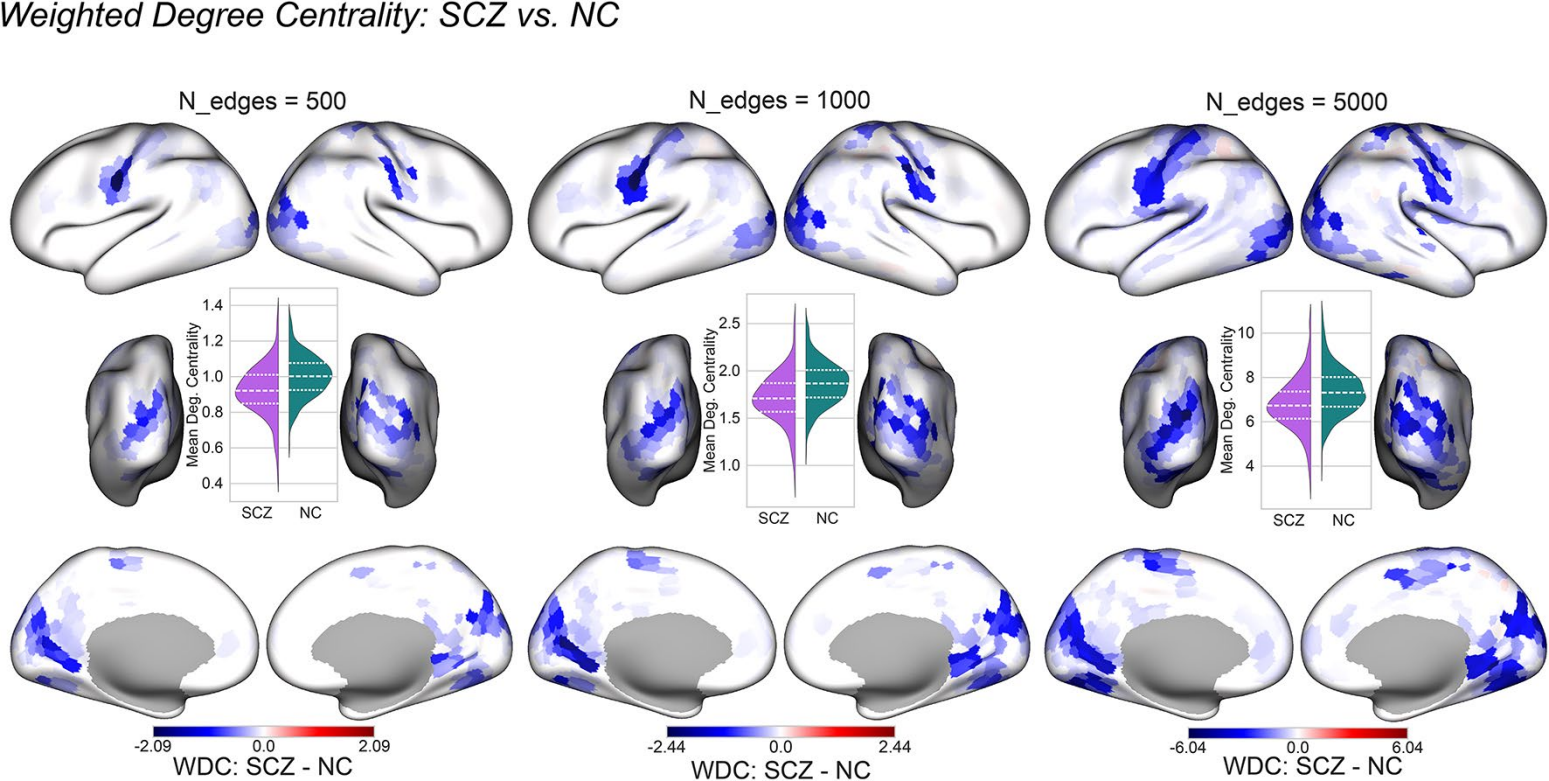
The difference in mean weighted degree centrality (WDC, averaged across subjects, Eq. 5) between the two groups for 500, 1000 and 5000 connectivity edges with the largest permutation feature importance. Inset violinplots display WDC averaged across regions for the two groups. Color bars denote the difference in WDC between SCZ and NC. SCZ: subjects diagnosed with schizophrenia, NC: neurotypical controls, WDC: weighted degree centrality.

For this analysis, we selected 500, 1000 and 5000 connectivity edges with the largest permutation feature importance and computed weighted degree centrality (WDC, Eq. 5. For each edge subset, we plotted the difference in group WDC between SCZ and NC (Fig. 6):

6 $$WDC_{diff} = WDC_{SCZ} - WDC_{NC}$$

WDC was overall lower in subjects with schizophrenia which is in line with previous accounts of lower overall functional connectivity characteristic of this disease^48–53^. This finding lends support to the dysconnectivity hypothesis of schizophrenia^54,55^. Furthermore, the spatial pattern illustrated in Fig. 6 indicated that the significant differences in WDC were concentrated in the primary regions for all edge subsets displayed here. Put differently, the edges with the largest permutation feature importance reflect connectivity strength in the primary regions, indicating that their connectivity profiles contribute the most to classification performance. The edges selected for this analysis can be viewed in Supplementary Material 1, 2 and 3 for 500, 1000 and 5000 most important edges respectively (the edges that were not selected are set to 0).

Additionally, we conducted a Mann–Whitney U test on WDC. The results align with the observations outlined above and can be viewed in Supplementary Fig. 2. However, we acknowledge that in the context of our feature selection procedure this test may be viewed as circular. Therefore, its results are to be interpreted with caution.

## Discussion

In this paper, we extend the effort to determine the optimal connectivity-based predictors of schizophrenia by attempting to benchmark connectivity-based features against each other for diagnosis prediction. To this end, we applied a feature selection workflow based on permutation feature importance to a large dataset comprising connectivity, macroscale cortical gradients and gradient dispersion. Our analysis revealed that, despite growing interest in cortical gradients and gradient dispersion as potential biomarkers of schizophrenia and psychosis^18,19,56^, functional connectivity holds superior predictive potential over its low-dimensional derivatives (Fig. 5B,C). Furthermore, we showed that this result remains consistent even when compared to the performance of a larger number of features of a different type. Specifically, the top 1% of connectivity features with the highest permutation feature importance performed better than the top 1% of all features and neighborhood dispersion features. Recently, an investigation similar to ours has been conducted for subject task performance^31^. Therein, the authors reported that individual parcellations achieved better predictions compared to cortical gradients. Although we employed a group parcellation, our findings align with this result.

Additionally, we demonstrated that the connectivity edges connecting the primary sensory regions have the largest permutation feature importance. This result indicates that variations in connectivity strength of these regions encapsulate critical information for distinguishing people with schizophrenia from neurotypical controls. This finding, however, does not discard the relevance of the gradients for the studies looking specifically into functional hierarchical variations. Regarding our use case, the diminished predictive capacity of the gradients could stem from the conservative matrix thresholding applied prior to dimensionality reduction. Future studies are needed to test this hypothesis.

To gain a deeper understanding of the connectivity patterns associated with schizophrenia, we conducted an exploratory analysis whereby we compared weighted degree centrality for the regions linked by the 500, 1000 and 5000 most important edges. Firstly, degree centrality appeared to be lower in individuals with schizophrenia, corroborating previous evidence of overall hypoconnectivity typical for the disorder^48–53^. Secondly, for the edges with the largest importance, these differences were predominantly concentrated in the sensorimotor, auditory, and visual cortex. These findings appear to contrast with the studies highlighting discrepancies predominantly in higher-order areas, such as the default mode network (DMN)^57–61^. However, the differences we observed need to be contextualized as the ones relevant for classification performance: while they may or may not constitute the neurological basis of schizophrenia, they are most informative for the differentiation of the two groups. Viewed from this perspective, our results resonate with the research demonstrating that across individuals the measurements are most reliable in the primary, unimodal areas^62,63^. In addition, one study reported more accurate surface registration for the primary sensory areas^64^. It has been postulated that this stability can be attributed to the fact that the primary areas are phylogenetically the most ancient cortical areas and are therefore considered as evolutionary anchors around which most of the cortical expansion in humans unfolded^63,,65–68^. The largest feature importance in the primary and most stable regions may indicate that the classifiers rely on those individual differences which are replicable across individuals of the same group. In such a setting, intra-individual variability cannot be accounted for. This shortcoming can be potentially addressed by studies focusing on datasets featuring hours of scanning time per individual^69–71^.

We were also interested in how the ranking of the 13 classifiers tested in this work evolved with the number of best features selected based on permutation feature importance. As depicted in Fig. 5D, some classifiers’ performance relative to each other fluctuated considerably depending on the size of the feature subset. Overall, no classifier outperformed others at all times for any number of features. It is a clear indication of the necessity of empirical testing of the candidate classifiers. However, it appears that as the number of features increases, the performance of linear classifiers — such as logistic regression and LDA—improves. Prior work offers insight into why linear classifiers would outperform non-linear approaches when the number of features disproportionately exceeds the number of training samples. Ardeshir et al.^72^ benchmarked the performance of support vector machine (SVM) and logistic regression in a high-dimensional (overparametrized) setting. The authors demonstrate that the solutions provided by SVM (which involves non-linear operations) and a linear classifier such as logistic regression coincide when the number of model parameters is large. This trend is the result of *support vector proliferation*^73^. In this scenario, SVM does not provide a performance boost compared to logistic regression. This phenomenon is in line with the evolution of our model ranking as a function of the number of predictors included in the model. Specifically, it is possible that adding more parameters to the model offsets the benefit of non-linear classifiers compared to their linear analogues. This account could explain the increase in the number of instances for higher numbers of features when linear models reached the top of the performance leaderboard.

Clinical neuroscientists frequently face the imbalance between sample size and the number of features. The choice of features with the highest predictive potential is critical in this line of work. With this problem in mind, we developed a pipeline which allowed us to select the most promising features from over one million candidates for diagnosis prediction. Based on PCA applied separately to each feature group and permutation feature importance, our approach enabled a systematic evaluation of the predictive capacity of each group, identifying connectivity as the winner. Notably, this result was achieved without testing all possible combinations of feature types which would be computationally challenging.

A number of limitations need to be noted. First, most subjects in our sample underwent scans lasting less than 6 minutes. Short scan time such as this prevented us from deriving individual parcellations^74^ which, as it has been demonstrated^74,75^, account of individual neuroanatomy and enrich functional connectivity with intra-individual variance. This variance was unavailable in our case since we relied on a group parcellation. In addition, prior investigations have articulated that cortical gradients are most discriminable across subjects when scan duration exceeds 30 minutes ^16^. Given that our circumstance did not align with this recommendation, a certain degree of caution is warranted when interpreting our findings.

Second, we did not account for the effect of medication in our study since the medication data were missing for a large number of participants diagnosed with schizophrenia. Excluding these subjects would have resulted in a drastically decreased sample size and, more importantly, in an increased imbalance of classes. We note however that even without these covariates, classification accuracy reached 80% and beyond for the features with the highest permutation feature importance.

Third, there were significant differences between both diagnosis groups and datasets in terms of age, sex distribution and framewise displacement. To address this limitation, we included all these variables as covariates in all models. Alternatively, cross-site harmonization could be applied to remove site-specific variance from the data. Various harmonization methods are available, ranging from linear regression to techniques more specific to MRI data such as ComBat^76,77^. However, all harmonization approaches known to date present a significant risk of reduction in sensitivity. That is, biologically relevant information may be regressed out along with site-related signals. In this work, we prioritized avoiding this risk.

Fourth, most classifiers in this study were fitted on datasets with more features than observations which increases the risk of overfitting. Nonetheless, we believe to have addressed this issue by employing a 10-fold CV for each classifier. In addition, we have also witnessed that for some classifiers test performance exceeded CV performance (Fig. 5C) which is not characteristic of overfitting.

Fifth, while permutation feature importance allowed us to select the most important features from multiple types, it was computed solely for logistic regression. Although feature rankings might vary depending on the model used to compute feature importances, we deem the results of this study robust for two reasons. First, based on cross-validation, logistic regression was rarely identified as the best model for feature sets containing fewer than 3000 features. That is, even though the features were ranked using logistic regression importances, this model did not outperform others for smaller feature subsets. Second, the usage of logistic regression for feature selection is a well-known strategy and proven effective in several domains^78,79^. Nonetheless, a comprehensive comparison of feature rankings generated by different models would be valuable and should be explored in future work.

In addition, we did not consider the issue of comorbidity, transdiagnostic phenomena across psychiatric disorders or subtypes of schizophrenia. While these aspects lie beyond the purview of the present study, it is imperative to acknowledge their critical significance. Inquiries into transdiagnostic classification, symptom prediction and subtyping hold potential to profoundly transform the paradigms governing the diagnosis and treatment of psychiatric and neurodevelopmental disorders. Indeed, as of late, the research looking into transdiagnostic effects^7,56,80–84^ and schizophrenia subtypes^85–87^ has garnered substantial momentum. We expect our work to assist future endeavors investigating these outstanding questions in benchmarking biomarker candidates.

The emergence of novel connectivity-based methods broadens our toolkit for predicting psychiatric disorders, introducing a necessity for empirical validation. Our findings indicate that functional connectivity outperforms its more recent, low-dimensional derivatives such as cortical gradients and gradient dispersion in predicting schizophrenia. Additionally, in this study, the connectivity within the primary sensory regions showed the highest discrimination capabilities, possibly due to the reduced anatomical and functional variability of those regions. We anticipate, however, that it is also informative for a broad spectrum of major psychiatric disorders. The exploration of this latter possibility warrants thorough examination in future work.

## Electronic supplementary material

Below is the link to the electronic supplementary material.

Supplementary Material 1.

Supplementary Material 2.

Supplementary Material 3.

Supplementary Material 4.

## Data Availability

The data used in this work are in public access; for COBRE: http://schizconnect.org/, SPBRS-1600: https://bicr-resource.atr.jp/srpbsopen/, and LA5c: https://openfmri.org/dataset/ds000030/. A user account on http://schizconnect.org/ is required to download COBRE. Access to SPBRS-1600 is conditioned upon completing the access application (https://bicr-resource.atr.jp/bicr_add/jsp/appForm.jsp) and signing a data sharing agreement. For questions about access to the SPBRS-1600 dataset, please contact decnef-db-admin@atr.jp or the corresponding author of this article: https://doi.org/10.1038/s41597-021-01004-8.

## Code availability

The code used to perform the analyses and to produce the figures featured in this study is available Github: https://github.com/victoris93/feature-selection-scz. All figures are generated using Seaborn (v. 0.13.2: https://seaborn.pydata.org/), Surfplot (v. 0.2.0: https://surfplot.readthedocs.io/) and Matplotlib (v. 3.9.3: https://matplotlib.org/) packages in Python. When necessary, the figures were compiled in panels using Affinity Designer (https://affinity.serif.com/, not open source).
