# Supplementary material for: A comparative machine learning study of schizophrenia biomarkers derived from functional connectivity
Source: Sci Rep. 2025 Jan 22;15:2849. doi: 10.1038/s41598-024-84152-2 (PMC11754439; doi:10.1038/s41598-024-84152-2)
Supplement: Supplementary file 4 — Supplementary Material 4. [file 41598_2024_84152_MOESM4_ESM.docx]

**A Comparative Machine Learning Study of Schizophrenia Biomarkers Derived from Functional Connectivity**

**Supplementary Methods 1: Scanning Parameters & fMRI Preprocessing**

The datasets used to compile the sample for this study are features in Table 1 along with scanning parameters at each site. The same preprocessing procedure was applied to all sites.

**Supplementary Table 1.** Summary of scanning parameters for each site. Datasets SWA, KUT, KTT, and UTO are part of a larger dataset, SPRBS-1600.

| **Dataset** | **COBRE** | **LA5c** | **SWA** | **KUT** | **KTT** | **UTO** |
| --- | --- | --- | --- | --- | --- | --- |
| **Site** | UNM Center for Psychiatric Research | UCLA | Showa university | Kyoto university | Kyoto university | University of Tokyo |
| **MRI Scanner** | SIEMENS TrioTim | SIEMENS TrioTim | SIEMENS Verio | SIEMENS TrioTim | SIEMENS Trio | GE Discovery MR750w |
| **Magnitic field** | 3T | 3T | 3T | 3T | 3T | 3T |
| **TR (s)** | 2 | 2 | 2.5 | 2.5 | 2 | 2.5 |
| **TE (ms)** | 29 | 30 | 30 | 30 | 30 | 30 |
| **Flip angle (deg)** | 75 | 90 | 80 | 80 | 90 | 80 |
| **Field of view (mm)** | 256 | 192 | 212 | 212 x 212 | 256 x192 | 212 |
| **In-plane resolution (mm)** | 3.75 × 3.75 | NA | 3.3 x 3.3 | 3.3125 x 3.3125 | 4.0 x 4.0 | 3.3 |
| **Slice thickness (mm)** | 3.5 | 4 | 3.2 | 3.2 | 4.0 | 3.2 |
| **Slice gap (mm)** | 4.55 | NA | 0.8 | 0.8 | 0 | 0.8 |
| **Number of slices** | 33 | 34 | 40 | 40 | 30 | 40 |
| **Total scan time** | 6:00 | 5:12 | 10:17 | 10:10 | 06:00 | 10:10 |

The following preprocessing steps were applied to each subject. A reference volume and its skull-stripped version were generated using a custom methodology of fMRIPrep. Susceptibility distortion correction (SDC) was omitted. The BOLD reference was then co-registered to the T1w reference using flirt (FSL 5.0.9^2^) with the boundary-based registration^3^ cost-function. Co-registration was configured with nine degrees of freedom to account for distortions remaining in the BOLD reference. Head-motion parameters with respect to the BOLD reference (transformation matrices, and six corresponding rotation and translation parameters) were estimated before any spatiotemporal filtering using mcflirt (FSL 5.0.9^2^). The scans were slice-time corrected using 3dTshift from AFNI 20160207^4^ (RRID:SCR_005927). The BOLD time-series (including slice-timing correction) were resampled onto their original, native space by applying the transforms to correct for head-motion. These resampled BOLD time-series will be referred to as preprocessed BOLD.

The BOLD time-series were resampled into standard space, generating preprocessed BOLD in MNI152NLin2009cAsym space. First, a reference volume and its skull-stripped version were generated using a custom methodology of fMRIPrep. Several confounding time-series were calculated based on the preprocessed BOLD: framewise displacement (FD), DVARS and three region-wise global signals. FD was computed using two formulations following Power (absolute sum of relative motions)^5^ and Jenkinson (relative root mean square displacement between affines)^2^. FD and DVARS were calculated for each scan using their implementations in Nipype (following the definitions by Power et al.^5^). The three global signals were extracted within the cerebrospinal fluid (CSF), the white-matter (WM), and the whole-brain masks. The frames that exceeded a threshold of 0.5 mm FD or 1.5 standardised DVARS were annotated as motion outliers. We regressed out the following artifacts: CSF, WM, and head motion confound timeseries and their quadratic derivatives. The full list of confound regressors as output by fMRIprep:

csf

white_matter

trans_x

trans_x_derivative1

trans_x_derivative1_power2

trans_x_power2

trans_y

trans_y_derivative1

trans_y_power2

trans_y_derivative1_power2

trans_z

trans_z_derivative1

trans_z_power2

trans_z_derivative1_power2

rot_x

rot_x_derivative1

rot_x_derivative1_power2

rot_x_power2

rot_y

rot_y_derivative1

rot_y_derivative1_power2

rot_y_power2

rot_z

rot_z_derivative1

rot_z_power2

rot_z_derivative1_power2

After initial preprocessing with fMRIprep, we further preprocessed the signal using Nilearn (Python). The timeseries were corrected for temporal drifts and trends and normalized (z-score). Finally, low- and high-pass filtering was applied at 0.01-0.08 Hz.

**Supplementary Methods 2: Cortical Gradients**

We provide screeplots of all 1000 gradients resulting from the PCA. We included the first 200 gradients in the main analysis since they cumulatively they explained 80% of variance on average.

***
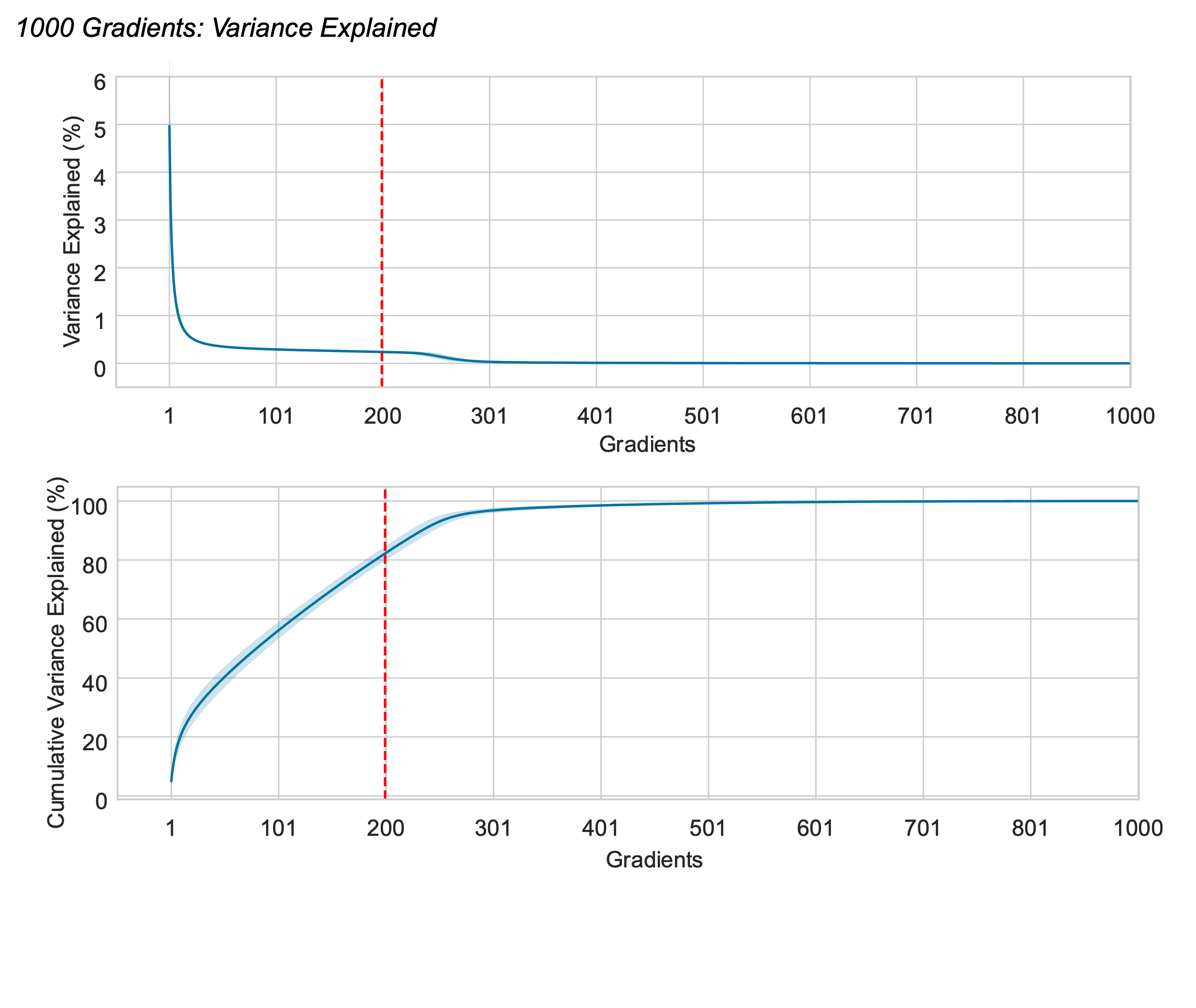
***

**Supplementary Figure 1.** Variance explained by each gradient (top) and cumulative variance explained (bottom) by all 1000 gradients. Dashed red line indicates the number of gradients chosen for this study: 200.

**Supplementary Results 1: Models Fitted on Principal Components of All Feature Types**

**Supplementary Table 2.** Model leaderboard, test metrics. Each model was fitted on 149 principal components of feature types. Every model included age, sex, site and framewise displacement as covariates. The models are ranked according to accuracy in the descending order. The model selected to compute permutation importances is in bold. In terms of fitting time, support vector machine (SVM) took the least time to fit, whereas Gradient Boosting classifier took the longest.

| **Model** | **Accuracy** | **AUC** | **Recall** | **Precision** | **Cohen’s Kappa** | **F1** | **Fitting Time (Sec)** |
| --- | --- | --- | --- | --- | --- | --- | --- |
| SVM - Linear Kernel | 0.8043 | 0.6935 | 0.4595 | 0.6939 | 0.4341 | 0.5528 | 0.38 |
| **Logistic Regression** | **0.7829** | **0.8251** | **0.5405** | **0.597** | **0.423** | **0.5674** | **0.49** |
| Gradient Boosting Classifier | 0.7687 | 0.7714 | 0.2703 | 0.6452 | 0.267 | 0.381 | 15.17 |
| K Neighbors Classifier | 0.7651 | 0.7369 | 0.3514 | 0.5909 | 0.304 | 0.4407 | 0.37 |
| Light Gradient Boosting Machine | 0.7473 | 0.8154 | 0.1892 | 0.56 | 0.1728 | 0.2828 | 0.38 |
| Naive Bayes | 0.7438 | 0.7698 | 0.3919 | 0.5179 | 0.2836 | 0.4462 | 0.37 |
| Random Forest Classifier | 0.7367 | 0.723 | 0.0 | 0.0 | 0.0 | 0.0 | 0.99 |
| Extra Trees Classifier | 0.7367 | 0.6326 | 0.0 | 0.0 | 0.0 | 0.0 | 0.78 |
| Dummy Classifier | 0.7367 | 0.5 | 0.0 | 0.0 | 0.0 | 0.0 | 0.36 |
| Ada Boost Classifier | 0.7331 | 0.7123 | 0.3243 | 0.4898 | 0.2283 | 0.3902 | 3.6 |
| Linear Discriminant Analysis | 0.6904 | 0.6534 | 0.5405 | 0.4301 | 0.2628 | 0.479 | 0.71 |
| Ridge Classifier | 0.6441 | 0.6282 | 0.5946 | 0.386 | 0.2185 | 0.4681 | 0.38 |
| Decision Tree Classifier | 0.6299 | 0.5578 | 0.4054 | 0.3333 | 0.108 | 0.3659 | 1.03 |
| Quadratic Discriminant Analysis | 0.5302 | 0.5422 | 0.5676 | 0.2958 | 0.4341 | 0.3889 | 0.68 |

**Supplementary Results 2: The Features with The Largest Permutation Feature Importance**

For each edge number of edges with the largest permutation feature importance, we conducted a Mann-Whitney U test. The results can be viewed in Supplementary Figure 2. Only the regions that survived the Bonferroni correction are displayed.


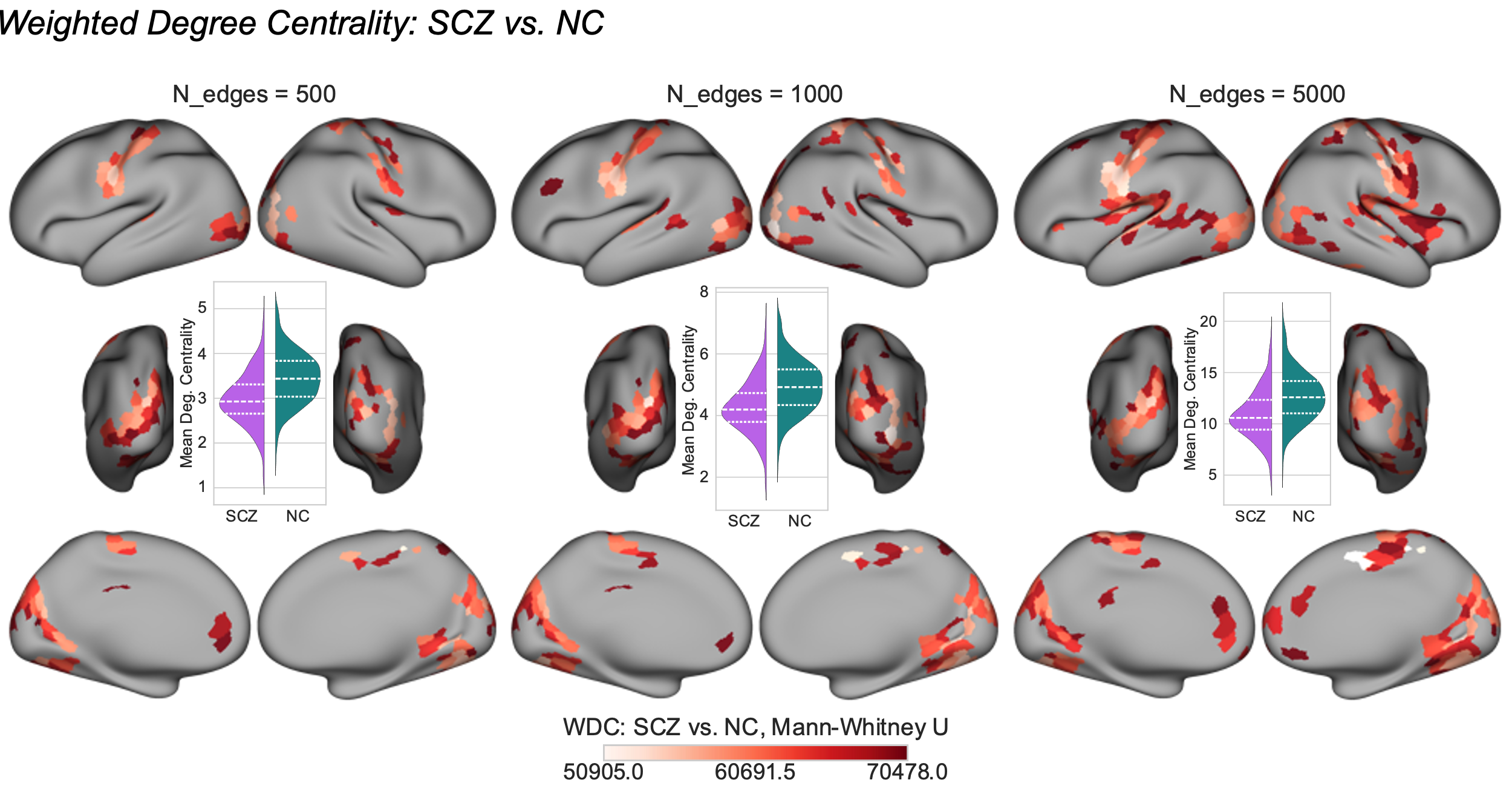


**Supplementary Figure 2.** Results of the Mann-Whitney U test for weighted degree centrality of 500, 1000 and 5000 connectivity edges with the highest feature permutation importance. Only the significant regions are displayed (α = 5 * 10^-5^, Bonferroni-corrected) Violinplot insets display the group difference in mean degree centrality across significant regions. SCZ: patients with schizophrenia, NC: neurotypical controls. Figure generated using Seaborn (v. 0.13.2: https://seaborn.pydata.org/), Surfplot (v. 0.2.0: https://surfplot.readthedocs.io/) and Matplotlib (v. 3.9.3: https://matplotlib.org/) packages in Python. All figures were generated by the first author.

**REFERENCES**

1. Tanaka, S. C. *et al.* A multi-site, multi-disorder resting-state magnetic resonance image database. *Sci Data* **8**, 227 (2021).

2. Jenkinson, M., Bannister, P., Brady, M. & Smith, S. Improved optimization for the robust and accurate linear registration and motion correction of brain images. *Neuroimage* **17**, 825–841 (2002).

3. Greve, D. N. & Fischl, B. Accurate and robust brain image alignment using boundary-based registration. *Neuroimage* **48**, 63–72 (2009).

4. Cox, R. W. & Hyde, J. S. Software tools for analysis and visualization of fMRI data. *NMR Biomed.* **10**, 171–178 (1997).

5. Power, J. D. *et al.* Methods to detect, characterize, and remove motion artifact in resting state fMRI. *Neuroimage* **84**, 320–341 (2014).
